# Dissociation of Axonal Neurofilament Content from Its Transport Rate

**DOI:** 10.1371/journal.pone.0133848

**Published:** 2015-07-24

**Authors:** Aidong Yuan, Linda Hassinger, Mala V. Rao, Jean-Pierre Julien, Christopher C. J. Miller, Ralph A. Nixon

**Affiliations:** 1 Center for Dementia Research, Nathan Kline Institute, Orangeburg, New York, United States of America; 2 Department of Psychiatry, New York University School of Medicine, New York, New York, United States of America; 3 Department of Cell Biology, New York University School of Medicine, New York, New York, United States of America; 4 Department of Psychiatry, McLean Hospital, Harvard Medical School, Belmont, Massachusetts, United States of America; 5 Centre de Recherche du Centre Hospitalier de l'Université Laval, Département d'anatomie et physiologie de l'Université Laval, Québec, Canada; 6 Department of Neuroscience, Institute of Psychiatry, Kings College London, London, United Kingdom; 7 Clinical Neurosciences, Institute of Psychiatry, Kings College London, London, United Kingdom; Texas Tech University Health Science Centers, UNITED STATES

## Abstract

The axonal cytoskeleton of neurofilament (NF) is a long-lived network of fibrous elements believed to be a stationary structure maintained by a small pool of transported cytoskeletal precursors. Accordingly, it may be predicted that NF content in axons can vary independently from the transport rate of NF. In the present report, we confirm this prediction by showing that human NFH transgenic mice and transgenic mice expressing human NFL Ser55 (Asp) develop nearly identical abnormal patterns of NF accumulation and distribution in association with opposite changes in NF slow transport rates. We also show that the rate of NF transport in wild-type mice remains constant along a length of the optic axon where NF content varies 3-fold. Moreover, knockout mice lacking NFH develop even more extreme (6-fold) proximal to distal variation in NF number, which is associated with a normal wild-type rate of NF transport. The independence of regional NF content and NF transport is consistent with previous evidence suggesting that the rate of incorporation of transported NF precursors into a metabolically stable stationary cytoskeletal network is the major determinant of axonal NF content, enabling the generation of the striking local variations in NF number seen along axons.

## Introduction

The axonal cytoskeleton is a network of NFs, microtubules, and actin filaments, extensively interconnected by cross-bridging proteins [1–3]. NFs are polymers of four subunits: heavy (NFH), medium (NFM), light (NFL) and either alpha-internexin in the CNS [4, 5] or peripherin in the PNS [6]. How NF protein assemblies undergoing transport are related to the NF network viewed ultrastructurally has been a matter of debate. The two NF population model states that NF precursors undergoing slow transport are a minor pool that maintains a large stationary metabolically stable NF network by a process of filament and subunit addition and exchange [7–10]. This model accounts for the ultrastructure of the axonal cytoskeleton as a network of interconnected fibrous elements, including NFs [11] and accords with evidence that NFs remain in place within axons for many months after their synthesis is turned off [12]. By contrast, in a single NF population model, NFs are considered to be one population continuously moving at a broad range of rates collectively corresponding to the slow wave of axonal transport [13]. The axonal NF cytoskeleton in this model would therefore be continuously turned over as it moves *en bloc* into nerve terminals and is replenished by newly synthesized NFs entering the proximal end of the axon.

Using genetic mouse models of NF subunit perturbation *in vivo*, we demonstrate that similar grossly abnormal axonal distributions of NF seen in two mouse models are associated with abnormal rates of NF transport in opposite directions. Moreover, in normal mice, the absolute number and regional density of NF varies dramatically proximally to distally along optic axons and we show that NF transport rate is constant along a length of optic axons where NF content varies 3-fold. These observations fulfill predictions of a two NF population model in which the rate of incorporation of a moving population of NF precursors into a stationary metabolically stable NF network is the principal determinant of the varying regional NF content along mature axons.

## Materials and Methods

### Ethics Statement

All experimental protocols were approved by the New York University and Nathan Kline Institute IACUC Committees in conformance with Public Health Service (PHS) policy on the humane care and use of laboratory animals and the NIH Guide for the Care and Use of Laboratory Animals. Mice were sacrificed by decapitation with previous anesthesia using isoflurane.

### Generation of knockout animals

Adult male or female mice of the C57BL/6J strain, aged 3–4 months at the time of injection or sacrifice, were used in all experiments. Mice were housed at 23°C on a 12-h light-dark cycle and were maintained on Lab Chow (Purina Mills, Gray Summit, MO) supplied at libitum. Production of NFH knockout has been previously described (HKO) [14]. Southern blot screening of knockout mice was performed as previously described [4].

### Generation of transgenic mice

Production of human NFH transgenic (hNFH tg) mice [15] and human NFL Ser55 transgenic (hNFL Ser55 tg) mice (harbouring a phosphorylation mutant NFL transgene in which Ser55 within the head domain of NFL was mutated to Asp so as to mimic permanent phosphorylation) was described before [16].

### SDS-PAGE and immunoblot analysis

Protein concentrations were determined with bicinchoninic acid (BCA) assay (Sigma-Aldrich, St. Louis, MO). SDS-PAGE was performed according to Laemmli [17]. Separated proteins were transferred to nitrocellulose membranes (Millipore, MA). Blots were probed with primary antibodies, followed by an alkaline phosphatase-conjugated secondary antibody (12500-fold dilution, DAKO, Glostrup, Denmark). The reaction was developed using a BCIP/NBT phosphatase system (KPL, Gaithersburg, MD) as described previously [18–20]. Optic pathways from knockout and wild-type controls were homogenized in 250μl μl of buffer (SDS buffer) containing 25 mM sodium phosphate (pH 7.2), 5 mM EGTA, 1% SDS, and 1 mM phenylmethylsulfonyl fluoride and protein concentration was measured with the BCA assay (Sigma-Aldrich, St. Louis, MO). Loading onto gels was normalized to total protein of the optic axons.

### Antibodies used

Antibodies used were mAbs to NFL (NR4), NFM (NN18) and NFH (N52) (Sigma Chemical Co.). Antibodies also used were mAbs to human NFH (OC95) and human NFL (DP5-112, Chemicon) and to both human and mouse NFH (N52) (Sigma Chemical Co.) and NFL (24.1) [21].

### Electron microscopy

Mice were anesthetized with halothane gas and the tissue was fixed through intracardial perfusion with 4% paraformaldehyde, 5% glutaraldehye in 0.1 M PBS, pH 7.4, at room temperature. The optic nerve was dissected and processed as described before [22], segmented in 1.2-mm pieces, cleared in propylene oxide, and embedded in Medcast (Ted Pella, Inc.). Ultrathin sections were collected and the section containing the initial portion of the retinal excavation was then used as a standard reference point. Ultrathin sections were stained with uranyl acetate and lead citrate and examined in an EX electron microscope (model JEM1200; JEOL) at 80 kV.

### NFs quantification

Between 120 and 383 retinal ganglion cell axon profiles were selected at each axonal level for analyses from the larger population of profiles solely on the basis that NF appeared unequivocally in cross-section throughout the axoplasm unless vesicular organelles occupied more than 50% of the cross sectional area (<5% of axons were excluded on this basis). All regions of the optic nerve section were used in obtaining sample profiles and all axons meeting the minimal inclusion criterion were analyzed. Axons were sampled in a caliber distribution that was identical to that in the total fiber population in optic nerves. NFs were counted from randomly assorted photomicrographs at 80,000x by a single investigator. Repetitions were accurate to ± 2.5%.

### Isotope injections

Adult mice were anesthetized with 13–20 μl g^-1^ body weight of Avertin (0.5 g tribromoethanol and 0.25 g of 2-methyl-2-butanol in 39.5 ml of distilled water) and received 0.30 μl of phosphate-buffered normal saline (pH 7.4) which contained 50 μCi of L-[^35^S] methionine (specific activity 1175 Ci /mmol purchased from Perkin Elmer Life Science Inc. (Boston, MA). Injections were made into the vitreous of each eye with a glass micropipette (70–100 μm) apparatus.

### Axonal transport studies

Since NFs and many of other proteins are non-uniformly distributed along nerves [23, 24], the distribution of a given labeled NF subunit is analyzed by comparing its amount in consecutive segments of nerve of equal length [25–27]. The retinal ganglion cells of adult knockout, transgenic mice and their wild type controls were radiolabeled by intravitreal injection. The primary optic pathway consists of the optic nerve, the optic chiasm, and part of the optic tract extending to, but not including, terminals in the lateral geniculate nucleus. Three to forty-two days after isotope injection, optic pathways from groups of 3 animals were dissected and cut into 8 consecutive 1 mm segments on a micrometer slide on dry ice. Triton-soluble and Triton-insoluble preparations from each segment were subjected to SDS-PAGE, electrotransfer of proteins, phosphorimaging, and autoradiography [28–30]. Because NFM peak was located at 1mm from the eye along optic axons a few hours after radioactive injection of WT mice and reached 2, 3, 4, 5, 6 and 7mm after 7, 14, 21, 28, 35 and 42 days, respectively, NFM peak transport rates were calculated by two methods: one including the first segment (axon initial segment) and the other excluding first segment. For example, at 7 days, NFM peak rates were 0.28 mm/day including first segment (2 mm divided by 7 days) and 0.14 mm/day excluding first segment (1 mm divided by 7 days). At 14 days, NFM peak rates were 0.21 mm/day including first segment (3 mm divided by 14 days) and 0.14 mm/day excluding first segment (2 mm divided by 14 days). Similarly, at 21 days, NFM peak rates were 0.19 mm/day including first segment (4 mm divided by 21 days) and 0.14 mm/day excluding first segment (3 mm divided by 21 days).

### Statistical analysis

Sample sizes were chosen according to the standard practice in the field. Significance was determined using unpaired two-tailed Student’s *t*-test or the Mann-Whitney test. The variance is similar between the groups that are statistically compared.

## Results

### NF content and distribution vary independently of NF axonal transport rates in transgenic mouse models

**Fig 1:**
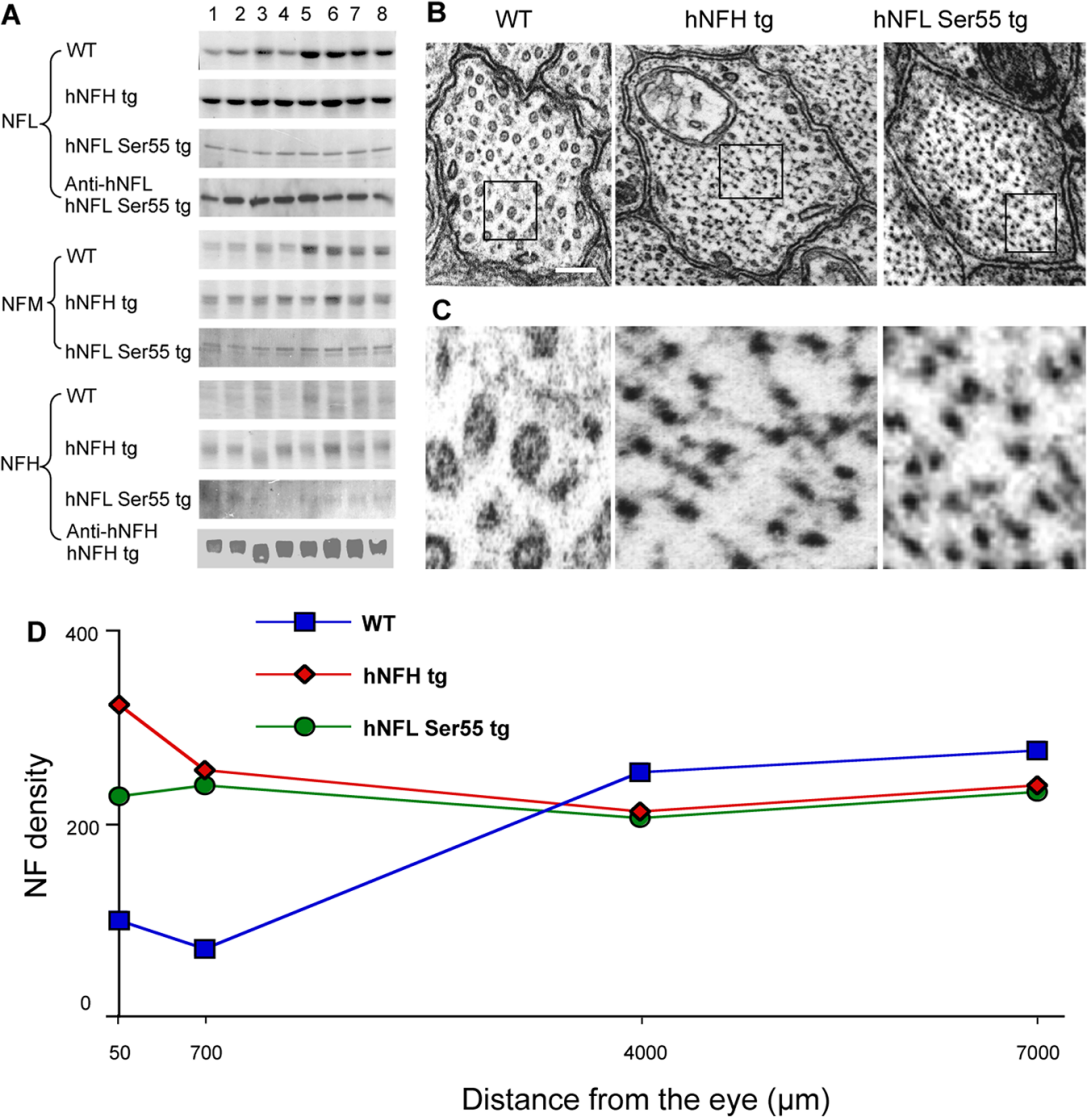
Two genetic mouse models exhibit similar patterns of abnormal axonal NF accumulation. (**A**) shows Coomassie Blue-stained gels of NFL, NFM and NFH distribution from 8 segments of optic pathway of WT, hNFH tg and hNFL Ser55 tg mice and also the distributions of hNFH and hNFL recognized by anti-human NFH specific antibody in hNFH tg and anti-human NFL specific antibody in hNFL Ser55 tg, respectively. Cross-sections of axons at the 50μm level of the optic nerve shows increased NF number in hNFH tg and hNFL Ser55 tg mice (**B**) and higher magnification of marked areas in B (C). Scale bar, 100nm. Average NF density measured in optic axons at levels 50, 700, 4000, and 7000 μm from the retinal excavation in 4 month old hNFH tg, hNFL Ser55 tg and WT mice (**D**). In each genotype of mice, 996 to 1377 axons in a size distribution matching that of the entire caliber distribution in the optic nerve axons were analyzed.

We analyzed the NF distribution along optic axons biochemically and by ultrastructural morphometry in relation to the *in vivo* axonal transport rates of pulse-radiolabeled NFs in mice overexpressing human NFH (“hNFH”) at a level 2-fold higher than endogenous levels [15] and in mice expressing human NFL in which Ser55 is mutated to Asp55 (“hNFL Ser55”) [16]. In the latter mouse model, 20% of the total NFL in brain is transgene-derived [16]. We first analyzed the longitudinal distribution of axonal NF proteins in 8 consecutive 1.1mm segments of the optic pathway after these proteins were separated from cytoskeletal extracts by SDS-PAGE and stained with Coomassie Blue (Fig 1A). Consistent with previous studies, the density of NF subunits along optic axons in WT mice increased 2–3 fold proximally to distally. By contrast, NF subunits distributed relatively uniformly along optic axons in both hNFH tg and hNFL Ser55 tg mice, reflecting an abnormally large size of the NF network at proximal levels of optic axons. Immunostaining of a companion blot with a monoclonal anti-human NFH specific antibody (OC95) confirmed a similar uniform distribution of human NFH along the optic pathway in hNFH tg mice (Fig 1A). A human-specific monoclonal antibody to NFL (DP5112) demonstrated uniform levels of mutant human NFL Ser55 (Asp) along the optic pathway in hNFL Ser55 tg mice (Fig 1A).

**Fig 2:**
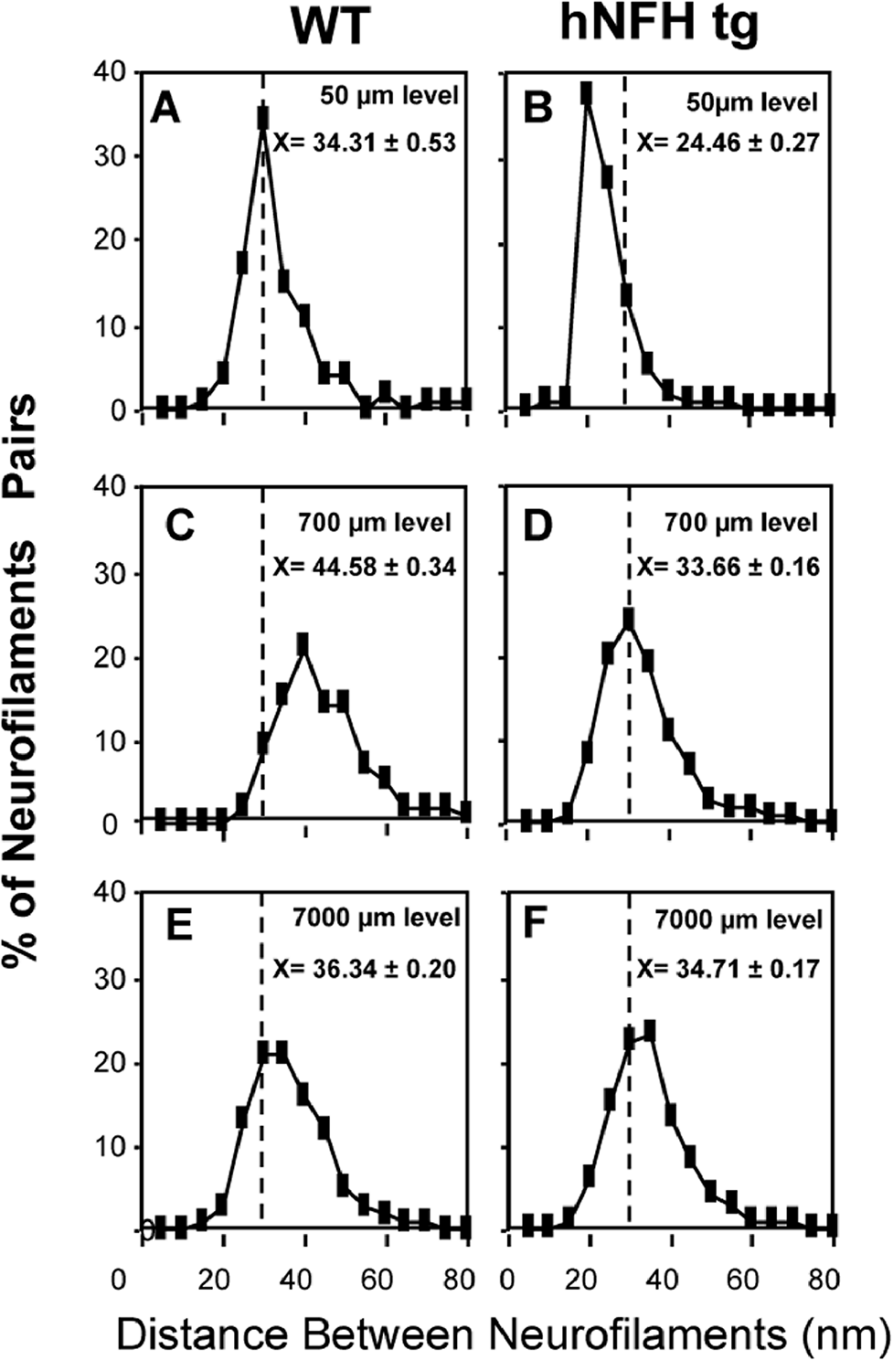
Relationship of the number of NFs to the cross-sectional area at two levels (50 μm and 700μm) along the optic nerve in WT (A, C) and hNFH tg mice (B, D). Region-specific shifts in inter-neurofilament spacing in hNFH tg mice (F, H, J) compared with WT controls (E, G, I) at the 50 μm, 700 μm and 7000 μm levels of the optic nerve. The nearest neighbor distance was calculated for every NF in axons of calibers representative of those in the total axonal population at the 50 μm, 700 μm and 7000 mm levels of the optic pathway.

To examine NF content and distribution in WT and Tg models, we next carried out morphometric analyses of NF number and axon caliber on >16,000 optic axons at proximal (50 μm, 700 μm), intermediate (4000 μm), and distal (7000 μm) levels of the optic pathways from adult transgenic and control mice. At each level, axons were selected randomly, but in approximately equal numbers, from each quadrant of the cross section. Electron micrographs of axonal cross-sections at the proximal level of optic nerves (50 μm from the retinal excavation) (Fig 1B and 1C) revealed substantially more numerous NF and a higher packing density in hNFH tg and hNFL Ser55 tg mice. Morphometric analysis to determine the distance between a NF and its nearest neighbor in hNFH tg and WT mouse axons indicated smaller interneurofilament spacing at the 50μm and 700μm levels of the optic pathway in the hNFH tg axons and a normalization of the distance at the more distal level (7000 μm) (Fig 2). Despite the higher packing density at proximal levels of hNFH tg axons, a normal shift toward higher interneurofilament spacing occurs in the network present at distal axonal levels (Fig 2E and 2F), indicating that this larger population of NF in hNFH tg mouse axons behaves normally with respect to NF side-arm phosphorylation [31].

EM morphometric analyses of NF density in WT mice demonstrated a striking non-uniform distribution of NF along optic axons, with a nearly 3-fold higher NF density at the most distal level (7mm) compared to that at the most proximal axon levels (50μm, 700μm). By comparison, NF densities in hNFH tg mice at these proximal levels were each elevated more than 2 fold in both hNFH and hNFL Ser55 mice although, at more distal axon levels (4–7mm), NF density normalized to WT values (Fig 1D). Thus, despite different genetic alterations, hNFH tg and hNFL Ser55 Tg mice displayed quite similar abnormal longitudinal NF distributions.

**Fig 3:**
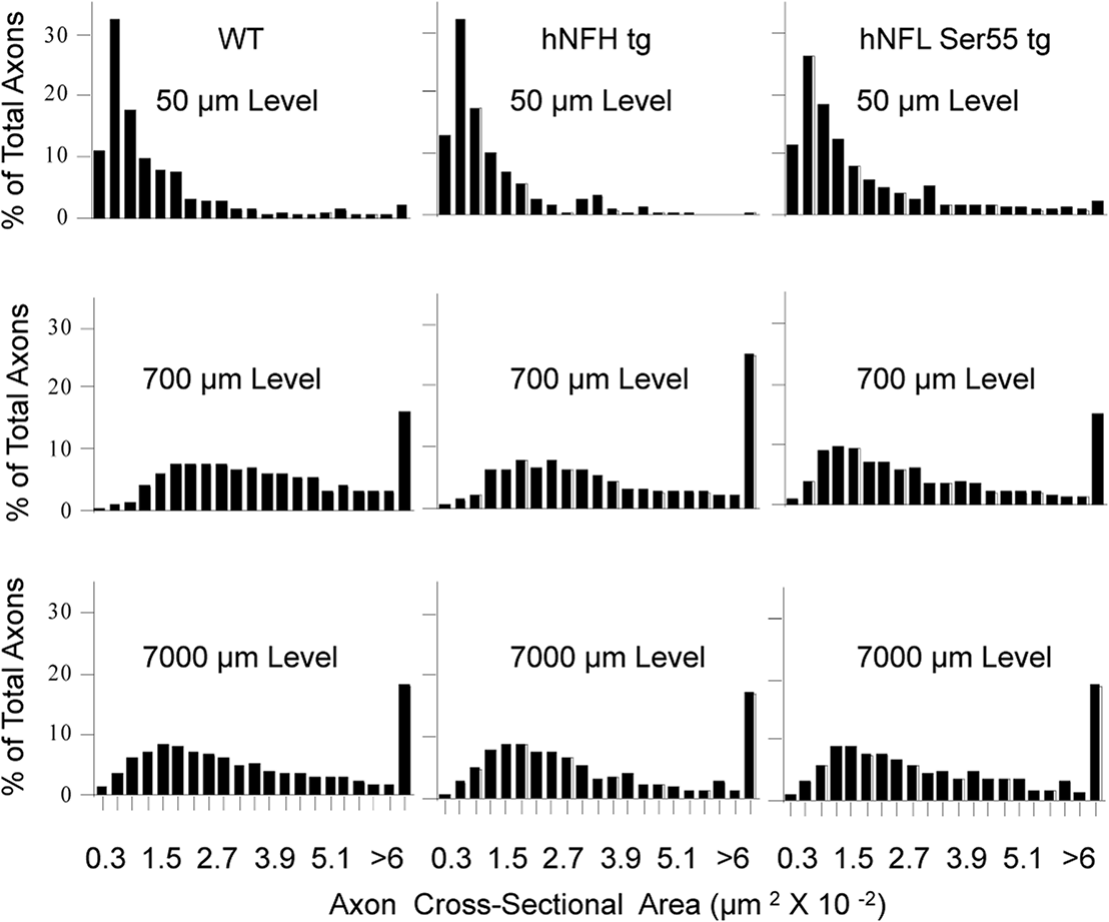
The percentage of axons relative to the total axonal population analyzed is plotted as a function of axon cross-section area. A total of 12,843 axons were analyzed at 50, 700, 4000 and 7000μm from the eye (n = 5 for each genotype).

Morphometric analysis of axon caliber distributions revealed, as previously described [22], that axons expand markedly in caliber (average 3-fold increase) between 50μm and 700μm from the retinal excavation. Thus, in WT mice, the calculations of NF density underestimates the extent of the proximal to distal increase of absolute NF number, which is >6 fold between the 50μm and 7mm levels. The caliber distributions of optic axons in hNFH and hNFL Ser55 tg mice were similar to that in WT mice, indicating that the large difference in axonal NF density pattern between the two tg mouse models and the WT mice was principally attributable to changes in NF number rather than axon caliber (Fig 3).

**Fig 4:**
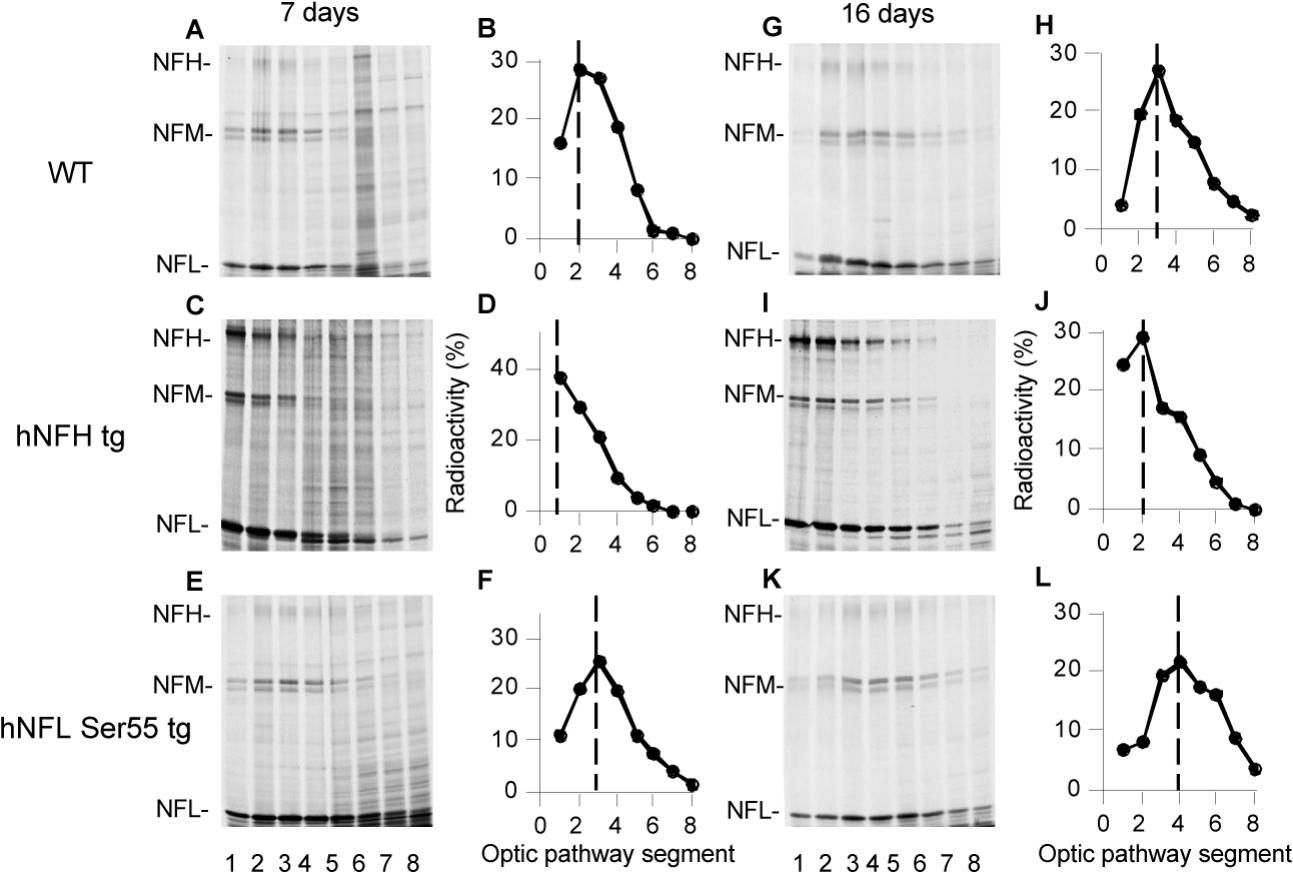
NF transport rates differ in mice exhibiting the same pattern of abnormal NF accumulation along axons. NF transport rate was determined by intravitreal injection of radiolabeled 35S-methionine into wild-type controls (**A-B, G-H**), hNFH tg (**C-D, I-J**) and hNFL Ser55 tg mice (**E-F, K-L**). At 7 and 16 days after pulse labeling, optic pathways were cut into consecutive 1.1mm segments and fractionated into cytoskeleton and soluble fractions with a Triton X-100–containing buffer. Fractionated proteins were separated on 5–15% SDS-polyacrylamide gels, transferred to nitrocellulose, and visualized by x-ray film and phosphorimaging. (**B, D, F, H, J, L**) Distributions of NFM were quantified despite nearly identical NF distribution (**Fig 1D**). NFM transport was significantly slowed in hNFH tg mice (**D**, **J**) but not in hNFL Ser 55 tg mice (**F**, **L**). Despite marked differences in NF distribution between hNFL Ser 55 tg and WT mice, NF transport in the tg model is unchanged or slightly faster (**F**, **L**). NFM peaks are denoted by dashed lines.

To determine how axonal transport may be affected in the above mouse models, we injected groups of hNFH tg, hNFL Ser55 tg, and WT mice intravitreally with [^35^S] methionine to pulse-radiolabel retinal ganglion cell proteins. The distribution of newly synthesized NF proteins was then analyzed at 7 and 16 days after injection in consecutive 1.1 mm segments of the optic pathway by autoradiography after SDS-PAGE. Despite similar NF distributions along axons, the transport of NF proteins relative to that in WT mice was substantially slower in hNFH tg mice but was modestly faster in hNFL Ser 55 tg mice (Fig 4). The NFM peak advanced to the second segment in WT mice at 7 days (Fig 4A and 4B), indicating a rate of about 0.28 mm/day (2 mm divided by 7 days), as previously reported [28, 29]. By contrast, in hNFH tg mice, the NFM peak was still at the first nerve segment, indicating a rate of 0.14 mm/day (1 mm divided by 7 days), (Fig 4C and 4D) whereas in hNFL Ser 55 tg mice the NFM peak advanced to the third segment. Rate differences were evident at 16 days postinjection when the NFM peak in WT mice moved to the third segment (3 mm divided by 16 days = 0.19 mm/days) (Fig 4G and 4H), whereas in hNFH tg optic nerves, the NFM peak had only moved to the second nerve segment (2 mm divided by 16 days = 0.13 mm/days) (Fig 4I and 4J) but had reached the fourth segment in hNFL Ser 55 tg mice (4 mm divided by 16 days = 0.25 mm/days) (Fig 4K and 4L). The substantial differences in axonal transport rates of NF subunits between hNFH tg and hNFL Ser 55 tg mice contrast sharply with the similar axonal NF distribution patterns in these two mouse models. Moreover, these differences in transport rates cannot readily account for differences in the NF distributions between the two tg mouse models and that of WT mice.

### Markedly reduced axonal NF content but unaltered NF transport rate in HKO mice

**Fig 5:**
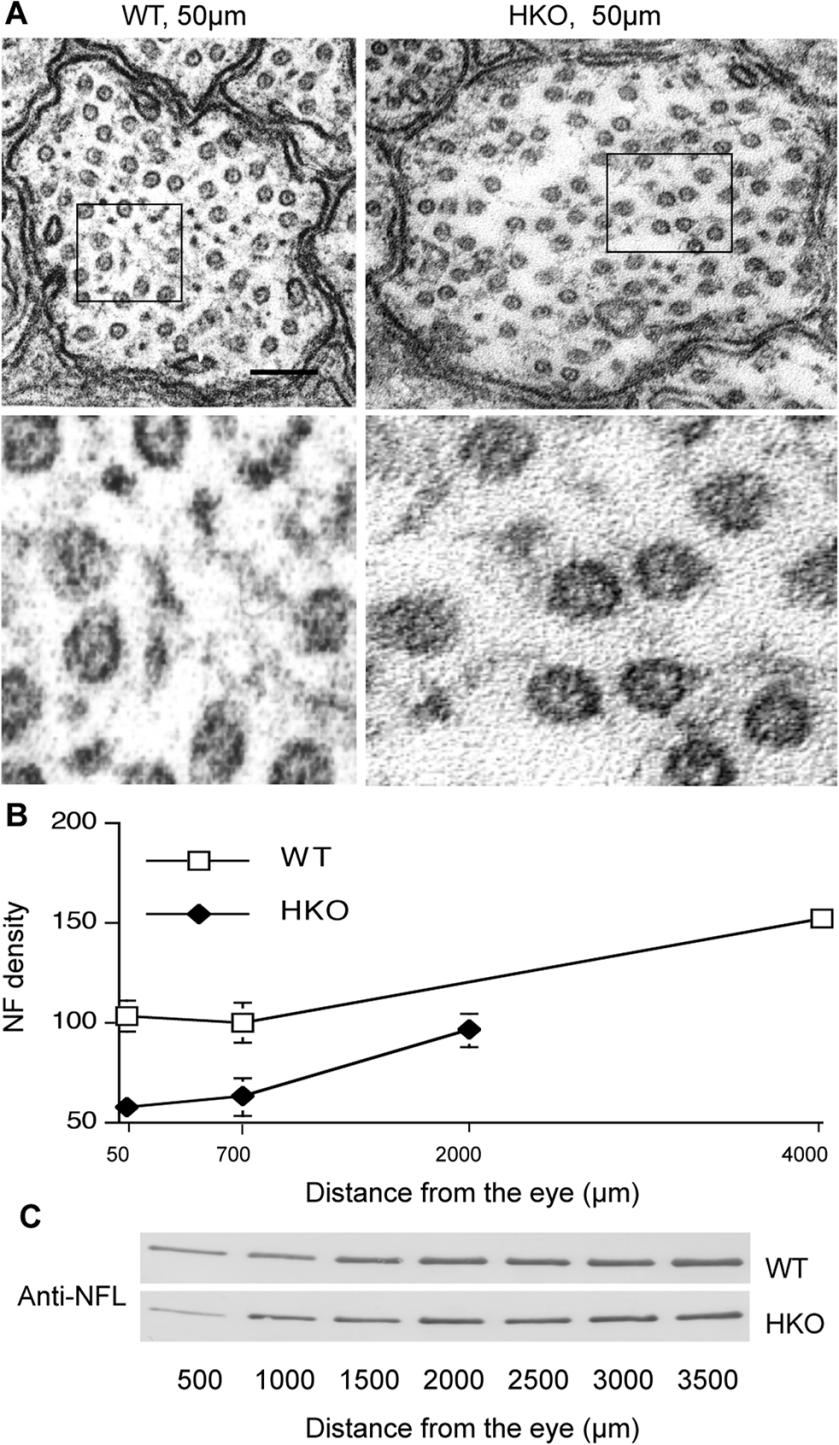
NF depletion in proximal optic axons in HKO mice. NF density is decreased at the proximal level (50μm) of optic axons in HKO mice compared with those in WT mice (**A, B**). The lower panel of A is the higher magnification of the marked areas in the upper panel. Scale bar, 100 nm. Decreased NFL in the most proximal segment of optic nerve (500μm) confirmed in immunoblot analyses of consecutive 500μm segments using an anti-NFL antibody along optic axons (**C**).

**Fig 6:**
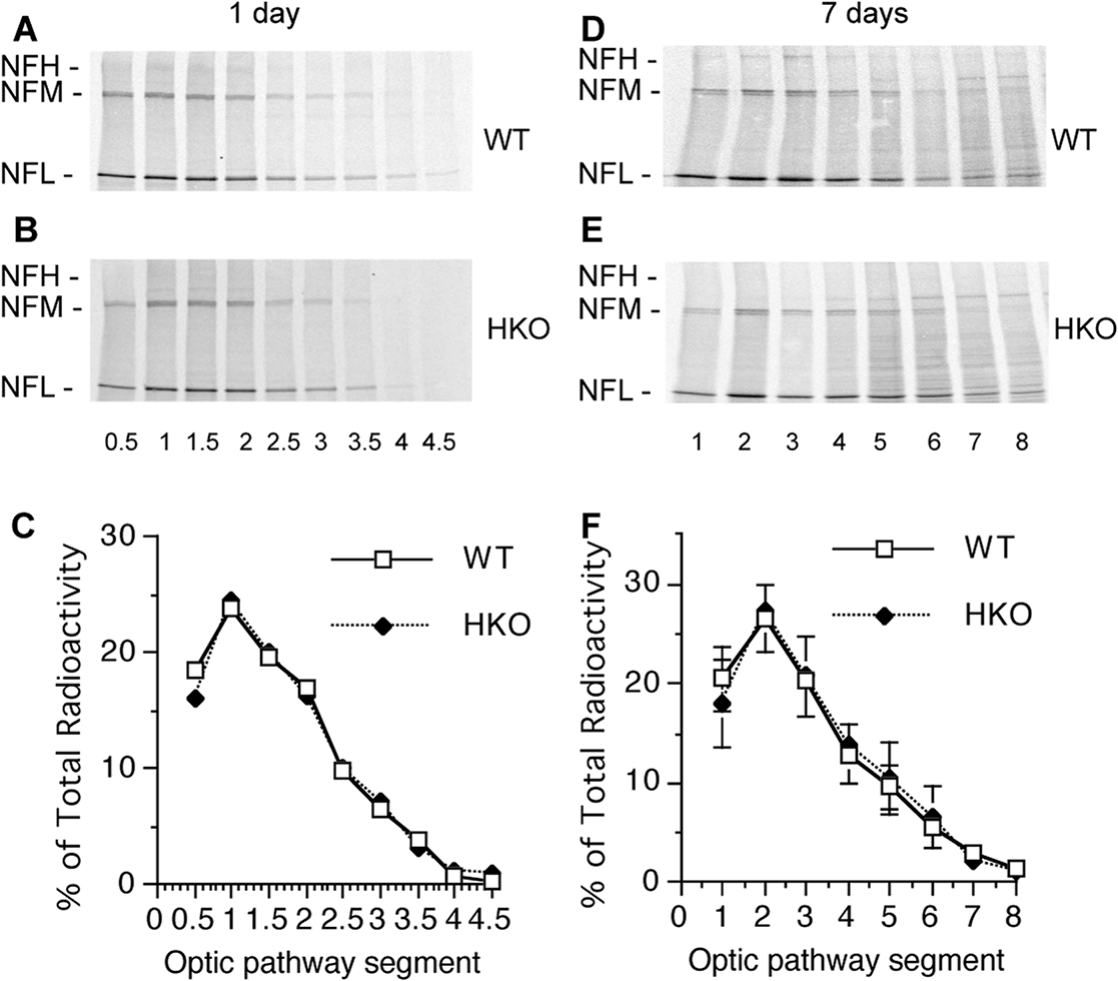
Normal NF transport rates in HKO mice with markedly altered NF distribution along axons. NF transport rate was determined as noted in Fig 4 legend except optic axons were dissected at 1 (**A, B**) or 7 days (**D, E**) after pulse labeling and optic segments were 0.5mm for **A-B** and 1mm for **D-E**. NFM transport rate is not significantly altered in HKO compared with WT mice at 1 (**C**) or 7 days (**F,** Mean ± SEM, n = 2).

The absence of transport rate abnormalities reported in mice lacking the NFH subunit (HKO mice) provided another opportunity to investigate the relationship of NF content to transport rate. Morphometric analyses of NF number in 9864 axons from 5 WT and 5 HKO mice were performed at optic nerve levels 50, 700 and 2000 or 4000 μm from the retinal excavation. At the 50 and 700 μm levels, NF density was lowered 44% (Mean ± SEM, P = 0.002, Student’s *t*-test) and 37% (Mean ± SEM, P = 0.0272, Student’s *t*-test), respectively, in HKO mice relative to that in WT mice (Mean ± SEM) (Fig 5A and 5B) consistent with an immunoblot analysis showing an approximately 50% reduction in NFL protein in a segment corresponding to the most proximal 500μm region of the optic nerve (Fig 5C). In this regard, reduced NF number was also observed in the proximal segment of phrenic nerve of the same mouse model [32]. NF density was less significantly lowered more distally, resulting in a proximal to distal differential of NF density in HKO that was even more extreme than that seen in WT optic axons (Fig 1). Despite this striking differential NF distribution in HKO mice, these mice exhibited a similar rate of NF slow transport as WT mice (Fig 6) with comparable ratio of total NFM radioactivity relative to that of actin (2.0 in HKO and 2.1 in WT controls at 1 day after injection).

Because the most dramatic changes in content in HKO mice were seen in the most proximal region of the optic nerve, we performed an even more discriminant analysis of NF transport *in vivo* than routinely used [4, 28–30] (Fig 6A and 6B) to possibly detect more subtle differences in transport rate at this axon level in HKO mice. In this analysis, we cut consecutive 0.5mm segments of optic nerve and analyzed the distributions of radiolabeled NF proteins at a one-day interval after injecting [^35^S] methionine. Even under these discriminating conditions, the NF transport rate in HKO mice was not detectably different from that of WT mice (Fig 6C), which was calculated here to be 0.21–0.25mm/day as in earlier analyses (Fig 4) [28, 29].

### The NFM transport peak moves at a constant rate along the myelinated WT optic axons

**Fig 7:**
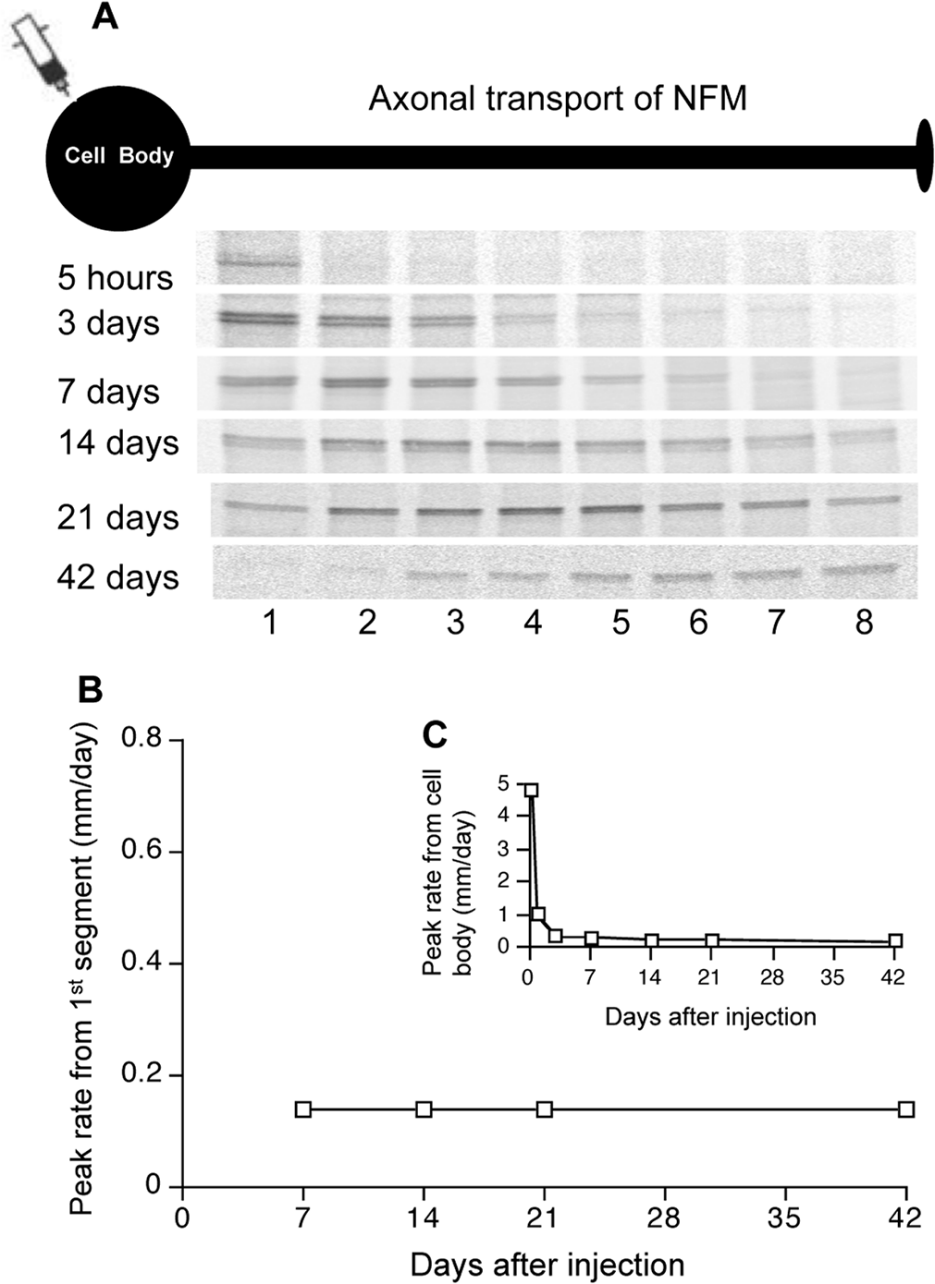
The behavior of radiolabeled NFM peak along optic axons. The radiolabeled NFM peak appeared within the first 1mm from the eye along optic axons a few hours after intravitreal injection of radiolabeled methionine and remained there for a few days with only the front of labeled NFM moving along the axons (**A**). NFM peak was transported at constant rate of 0.14 mm/day if measured specifically along the myelinated length of the optic nerve and tract to avoid influences from axon initial segment (**B**). If NFM transport rate was calculated from the cell body and including the axon initial segment, it appeared that NFM transport slows down along the axons (**C**).

**Fig 8:**
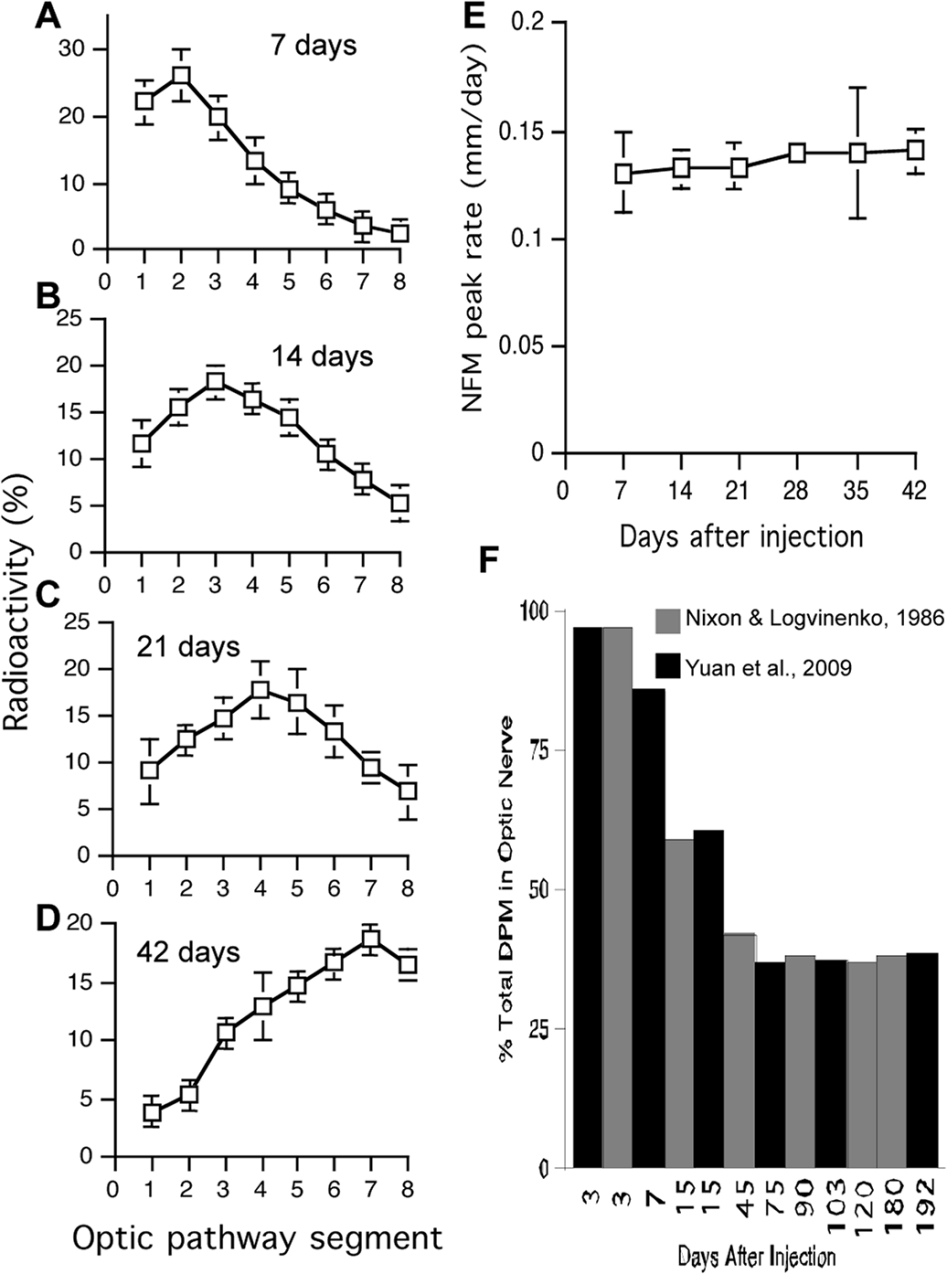
NFM transport rate remains constant along optic axons except the first segment. Contradicting a critical assumption of the Li et al. mathematical model, NF transport rates determined at 7 (**A**), 14 (**B**), 21 (**C**), 42 days (**D**) post injection as in Fig 4, show that NFM peak advance is constant along the optic axons [for example, 0.14 ± 0.02 mm/day (Mean ± SEM, n = 19) at 7 days; 0.14 ± 0.01 mm/day (Mean ± SEM, n = 14) at 14 days] (**E**) until the wave exits the optic window (**A**-**E**). (**F**) Percentage of NFM radioactivity (in dpm) in optic nerve (segments 1–4) relative to total NFM dmp in optic pathway (segments 1–8) in long-term labeling studies from two independent analyses [7, 9]. The constant percentage in optic nerve at 75-to-192 days indicates no net movement of NF containing labeled NFM. Total number of mice analyzed in both studies equals 262–302. Gray bar, Nixon and Logvinenko, 1986; black bar, Yuan et al. 2009.

**Fig 9:**
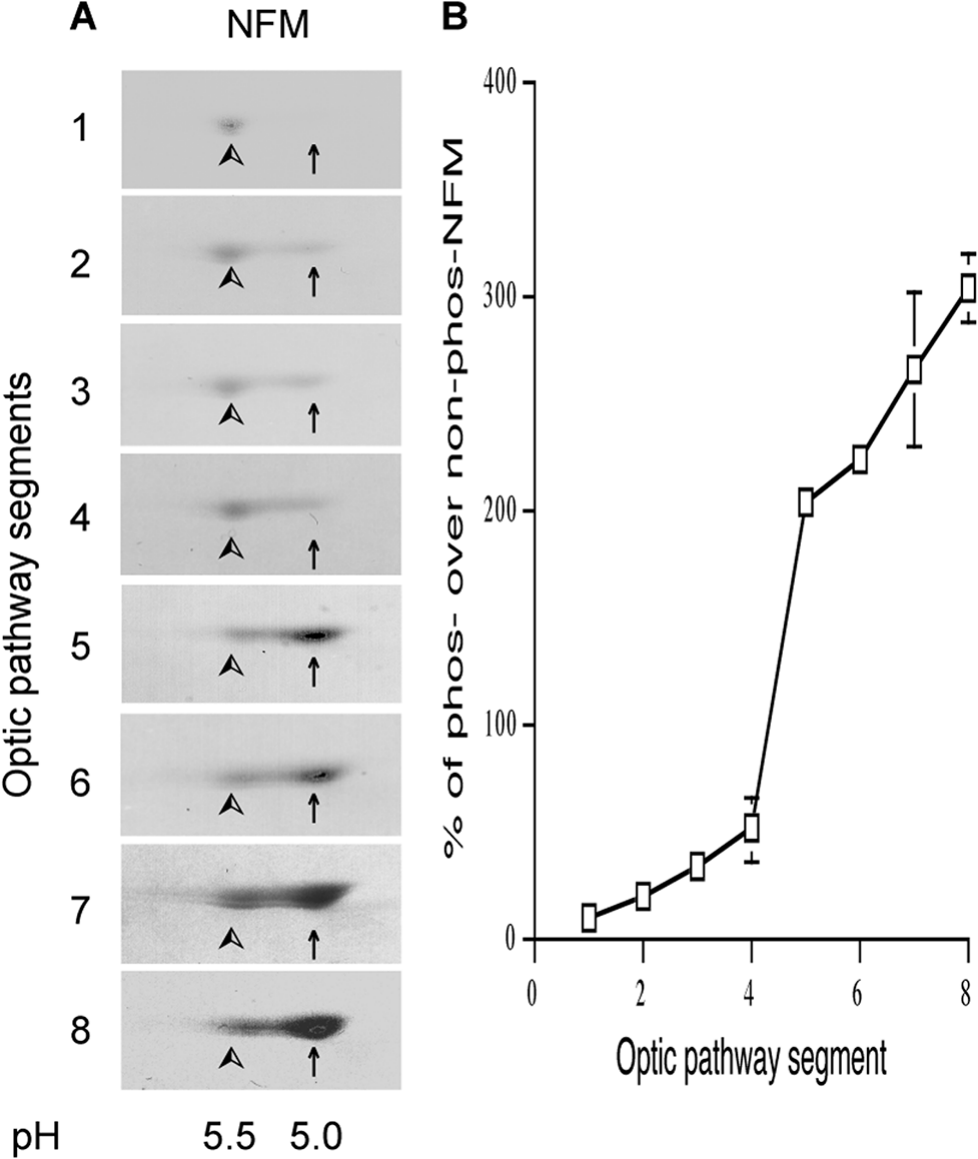
NFM phosphorylation increases along the optic pathway in a proximal-to-distal manner. Coomassie blue-stained 2D gels of Triton-insoluble fractions from consecutive 1 mm segments of the optic nerve and tract demonstrate that phosphorylated NFM (indicated by arrows, pI 5.0) shows proximal to distal increase (**A**). The ratios of phosphorylated NFM over non-phosphorylated NFM (indicated by arrowheads, pI 5.5) increase up to about 300% along optic pathway (Mean ± SD, n = 2, **B**).

Previously, investigators of axonal transport have used either the actual radiolabeled NF peak to calculate NF transport rate or the position of the “50^th^ percentile” of NF radioactivity as an imaginary NF transport peak to calculate transport rate. The latter method is less accurate because it does not take into account the radiolabeled NF proteins moved out of the analyzed window or the population of NF that have incorporated into the stationary NF network. Moreover, the most proximal unmyelinated portion of myelinated retinal ganglion cell (RGC) axons (“initial segment”) varies in length and the largest proportion of RGC has very short initial segments close to the retinal excavation. Because the axon initial segment may contain ribosomes [33] capable of translation of proteins and a uniquely high density of specific voltage-gated channels [33–35], we measured NF transport rates specifically along the myelinated 7mm length of the optic nerve and tract in WT mice to avoid influences from this unique portion of the optic axons. Supporting the atypical apparent transport rate in the axon initial segment, we observed that the radiolabeled NFM peak appeared within the first 1mm from the eye along optic axons a few hours after intravitreal injection of radiolabeled methionine and remained there for a few days with only the front of labeled NFM moving along the axons. If we calculated NFM transport rate from the cell body and including the axon initial segment as in some previous studies [36, 37], it appeared that NFM transport slows down along the axons (Fig 7C). By contrast, once radiolabeled NF moved out of the first segment, the NFM peak moved at a constant rate (Fig 7B) of 0.14 ± 0.02 mm per day (Mean ± SEM, n = 19) along the entire length of the optic axonal window until it reached the end of this window after 7 weeks (Fig 8A–8E). We also observed an increase of NFM phosphorylation along the optic pathway in a proximal-to-distal manner using 2-dimensional gel electrophoresis (Fig 9).

### Transported NFs that incorporate along axons do not redistribute over many months *in vivo*

In Fig 8F, we analyzed our data from two independent studies [7, 9] that describe the long-term fate of pulse-radiolabeled NFs in optic axons. Using the data from both studies, we calculated the proportion of NFM radioactivity in optic nerve (segments 1–4) relative to radioactivity in the whole optic axon window (segments 1–8). Proportions of labeled NFM declined progressively between 7 and 45 days, corresponding to the movement and ultimate exit of labeled NFM from the optic nerve. By contrast, the proportions of labeled NFM between 75-to-192 days remained constant, indicating that there is no net movement of this labeled NF population, consistent with previous evidence for distinct slowly transported and stationary populations demonstrated using other experimental approaches [7, 9, 12].

## Discussion

**Fig 10:**
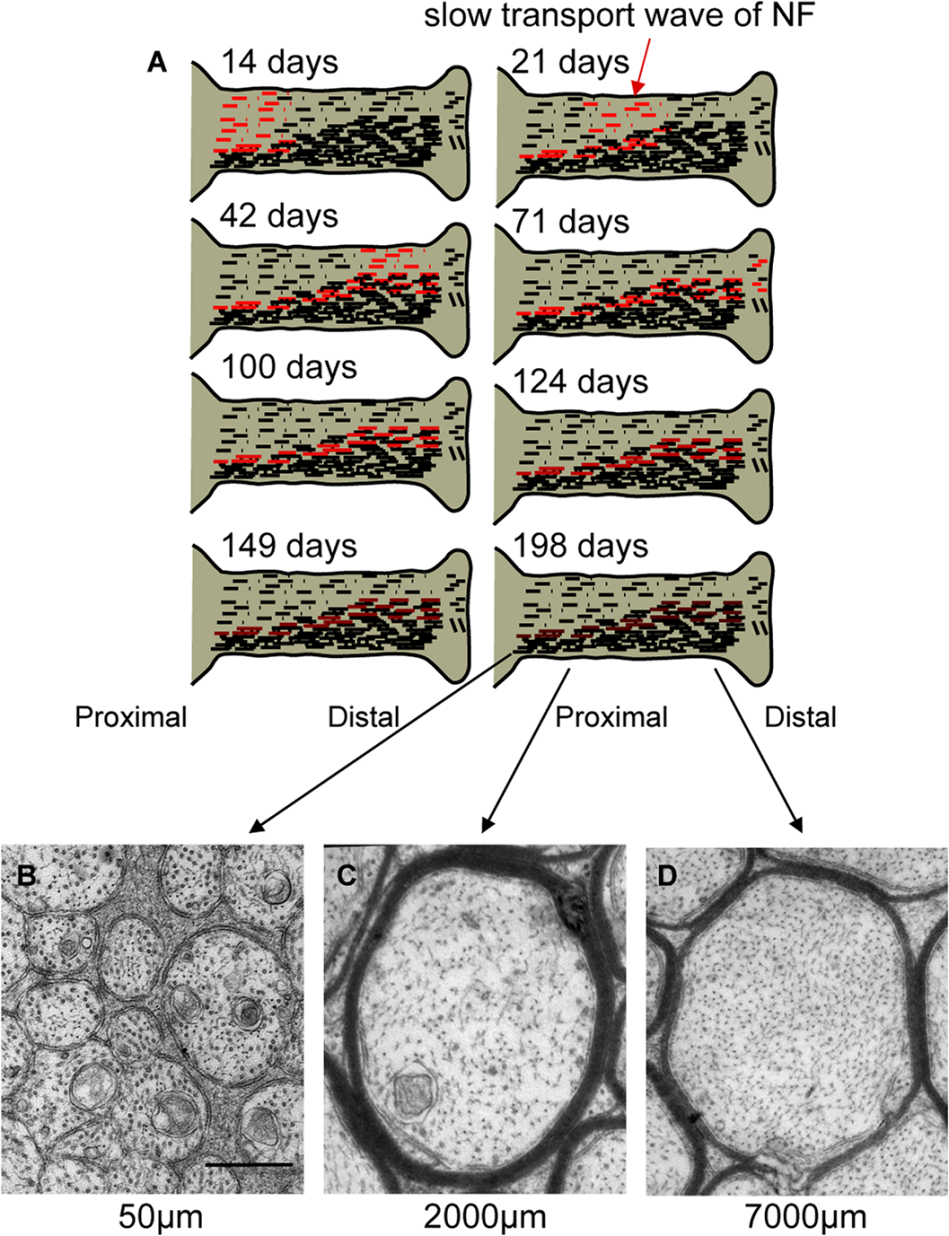
Model describing fates of newly assembled NF proteins in transport and formation of a non-uniform stationary axonal cytoskeletal network. **(A)** The radiolabeled sub-population of newly synthesized NF and protofilaments (red dotted lines and dots) is transported along axons within the slow wave of axonal transport at a broad range of rates averaging 0.14 ± 0.02 mm per day, and reflecting brief periods of rapid movement and longer periods of pausing (**E**). As they are transported, radiolabeled NFs are continually incorporated into the stationary NF network containing preexisting unlabeled NF (indicated as black filaments) (**A**) which turn over at a very slow rate (half-life > 83 days). The greater level of incorporation into the stationary network at distal axonal levels (Nixon and Logvinenko 1986) generates the proximal-to-distal gradient of increasing NF content along optic axons at steady state in mature neurons (**B**-**D**).

Our findings demonstrate that, under a range of experimental conditions, the content and regional distribution of NFs along axons can vary strikingly and independently of any commensurate change in NF transport rates. Consistent with these data are additional recent findings that deletion of the carboxyl terminal tails of NFM and NFH causes NF density to fall 6-fold proximally and 3-fold distally in the absence of any alteration in the NF transport rate or quantity of newly synthesized NF entering axons [38]. This sizable decline in steady state NF levels was associated with a selective decline in the amount of labeled NF protein incorporated into the stationary NF network and higher NF turnover [38](Fig 10). Moreover, our analyses of the studies on the long term fate of pulse-labeled NF proteins establish that, after the slow wave of transported NF proteins disappears from optic axons during the first 75 days after synthesis [7, 9], the labeled NF still remaining in axons displays no net movement (i.e., proximal to distal redistribution) over the next 4 months. This remaining stationary population of radiolabeled NF distributes in the same non-uniform pattern as the entire content of NF quantified by ultrastructural morphometry and turns over with a half-life exceeding 83 days [9] or even more slowly in peripheral nerves [12]. Local NF synthesis from reutilized labeled amino acids in axons is not a contributing factor to the long half-life [7], as further suggested by the complete loss of the NFM protein from optic axons when its transport is inhibited by deleting alpha-internexin, NFH and NFL [39]. How locally increased NF accumulation is achieved in hNFL Ser 55 tg mice despite a modestly *faster* NF transport rate is less clear at present. Previous studies suggest that phosphorylation of Ser55 does not, on its own, control assembly of NFL into intermediate filament arrays but might induce subtle changes in NF architecture [40], which could then promote the incorporation of labeled NF proteins into the stationary network.

The striking differences in NF content and non-uniform axonal distribution patterns seen in different NF mouse models, and the lack of relationship of these differences to changes in NF transport rate, are incompatible with a single NF population model in which NFs undergoing slow axonal transport compose the entire NF content of axons, as proposed in a mathematical modeling study of previously published data on mouse optic axons [13]. Key assumptions made in this mathematical analysis included slowing of NF transport along axons and co-migration on SDS gels of NFM subunits with contaminating SCb proteins, neither of which is supported by our present findings and earlier publications [4, 9]. Moreover, a more recent mathematical analysis concluded that existing NF transport data conform better with a two NF population model of the axon cytoskeleton than a single population model.

**Fig 11:**
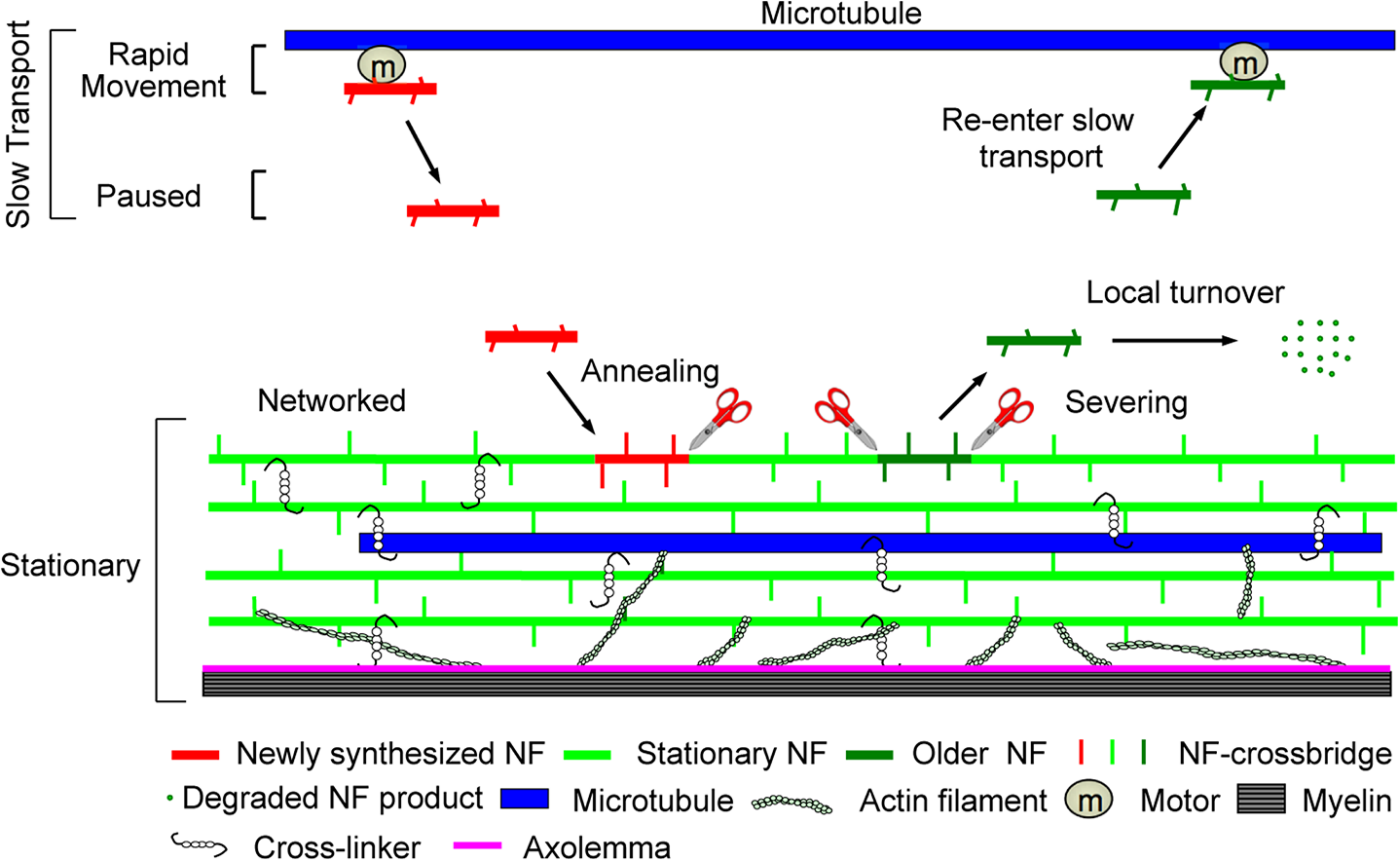
Model of NF behavior in axons. The first process is the slow transport of short NF or hetero-oligomer that is the net rate achieved from the many intermittent, rapid movements and “pauses”, possibly reflecting the reversible attachment of NF to a molecular motor moving on a microtubule. The second process is the active annealing and integration of short NF into a distinct stationary axonal cytoskeleton. Older NF may degrade locally through severing and proteolysis or by re-entering the pool of slowly transported NF.

Our observations in this report and other existing evidence on NF transport (Table 1) are best explained by a two NF population model in which the NF cytoskeleton is comprised of a small moving NF population that maintains a large long-lived stationary NF network (Fig 11). In this model, the regional content of NF along axons and the total NF content of axons are determined mainly by the rate at which NF in slow transport are incorporated into the stationary cytoskeleton and by half-lives of stationary NF that are exceptionally long and appear to be similar along optic axons [7]. Previous reports supported the hypothesis that NF content is controlled by the axonal transport of NF [41]. Our present findings are compatible with this view but emphasize that rate of incorporation of transported NF into the stationary cytoskeleton and the half-life of this structure rather than NF transport rate itself, are the key determinants of cytoskeleton size. NF subunits were previously shown to increase proximally to distally along the optic pathway [4] and here we observed a parallel gradient of NFM phosphorylation as previously seen in cultured neurons [42–44]. Since the tail domains of NFM and NFH form cross-bridges that inter-connect NFs and link them to other cytoskeletal components and membrane-bound organelles [45–48], their phosphorylation is implicated in regulating interaction with other elements of the cytoskeleton [49] and increasing NF half-life in the stationary cytoskeleton by blocking NF proteolysis [38, 50–52].

**Table 1:** Dissociation of NF content from NF transport rate. LKO, NFL knockout mice; MKO, NFM knockout mice; HKO, NFH knockout mice; HL-DKO, NFH and NFL double knockout mice; DTL, mice lacking NFH and NFM tail domains; hNFH tg, mice overexpressing human NFH; hNFL Ser55 tg, mice expressing human NFL mutated at Ser55 (Asp). GAN, giant axonal neuropathy.

*Animals*	*NF number*	*NF transport rate*	*References*
Wild-type mice	100%	Normal	[28]
LKO mice	Rare	No change	[28][59]
MKO mice	43% of WT in L5 ventral root	Increase	[4][60][61]
HKO mice	About 50% of WT at 50μm from the eyeball	No change	This study
HL-DKO mice	<10% of WT at 2mm from the eyeball	No change	[28]
DTL mice	10% of WT at 50μm from the eyeball	No change	[38]
hNFH tg mice	>200% of WT at 50μm from the eyeball	Decrease	[62], this study
hNFL Ser55 tg mice	>200% of WT at 50μm from the eyeball	Increase	This study
GAN in rats	40% of WT at 5mm from the eyeball	Increase	[63]

Despite their non-uniform distribution along axons, NFs appear to be transported at similar rates at different levels of optic axons in mice of a particular age. NFs are transported by kinesin and dynein motors along microtubules [53] and current evidence suggests that the major determinants of transport rate are the motor activity and the integrity of the tracks [54–58] Steric hindrance of moving NF by stationary structures may be among additional influences, given that axonal transport rates slow with maturation and aging as the content of stationary cytoskeletal elements increases [37]. Changes in the transport rate of the relatively small pool of moving precursor NF protein assemblies are expected to have relatively minimal impact on steady-state NF levels, as we have demonstrated here. Our results do not exclude changes in NF protein synthesis rate or entry into axons as additional determinants of steady-state NF levels, as may occur during neuronal development or regeneration when NF synthesis may be dramatically up-regulated. In mature axons, however, large changes in NF synthesis are not observed and none were noted in the mouse models in this study. The formation of a large metabolically stable stationary network in fully developed axons rather than a moving wave of NF continuously turning over NF at the synaptic terminals is expected to have strong advantages for neuronal energetics, given that NF can be as much as 13% of the total protein content of PNS nerves [6].
